# Microsurgical pineal cyst fenestration: A safe and effective treatment strategy in patients with symptomatic pineal cyst syndrome

**DOI:** 10.1007/s00701-025-06569-6

**Published:** 2025-06-30

**Authors:** Silvia Hernández-Durán, Xenia Hautmann, Marie-Luise Werner, Veit Rohde, Dorothee Mielke

**Affiliations:** 1 Department of Neurosurgery, University Hospital Göttingen, Robert-Koch-Str. 40, 37075 Göttingen, Lower Saxony Germany; 2 Department of Neurosurgery, University Hospital Augsburg, Stenglinstr. 2, 86156 Augsburg, Bavaria Germany

**Keywords:** Pineal cyst, Cyst fenestration, Cephalgia

## Abstract

**Purpose:**

Pineal cysts (PC) are benign cysts of the pineal gland (PG). Some patients with PCs have nonspecific symptoms such as headache, sleep disturbances, dizziness, and nausea. In cases of hydrocephalus or Parinaud syndrome, surgical removal of the cyst is the gold standard. However, even without evidence of these signs and symptoms, some patients are offered to undergo surgery. Regarding the indication, the surgical methodology and the clinical outcome in case of unspecific symptoms, evidence is insufficient.

**Methods:**

All patients undergoing microsurgical fenestration for PC from 2005 – 2021 were included in the analysis. A survey was carried out inquiring about preoperative symptoms, limitations in daily activities, medication use, and postoperative improvement. Preoperative radiological parameters were also evaluated. The primary endpoint of this study was symptom improvement at last follow-up, as assessed by our survey. Radiographic and clinical factors were evaluated for their correlation with symptom improvement. Secondary endpoints were surgical complications, such as surgical site infections (SSI), cerebrospinal fluid fistula (CSF fistula), intraoperative bleeding and mortality.

**Results:**

Forty-seven patients were included in the analysis. The follow-up period ranged from 5 to 176 months, with an average follow-up time of 84.13 months (about 7 years) post-surgery. Mean preoperative visual analogue scale (VAS) was 7 (SD 2.34), while postoperatively it was 1 (SD 1.91). The majority of patients (94%) showed a decrease in their VAS postoperatively. In a paired-sample t-test for the VAS, the postoperative improvement was statistically significant (*p* < .001). No mortality or severe adverse events were reported.

**Conclusions:**

Microsurgical cyst fenestration can yield symptom improvement in non-hydrocephalic patients with PC and unspecific symptoms. Further work is needed to validate both this surgical technique and indications therefor.

**Supplementary Information:**

The online version contains supplementary material available at 10.1007/s00701-025-06569-6.

## Introduction

Pineal cysts (PC) are benign entities located in the pineal gland (PG) [9]. According to large MRI studies, the prevalence of PCs in the population is 1- 37.5% [1, 9, 23]. A very recent MRI study showed the highest incidence of 37.5% with a female predominance and a predominant proportion (19.6%) of small PCs (< 0.5 cm). A very recent MRI study showed the highest incidence of 37.5% with a female predominance and a predominant proportion (19.6%) of small PCs (< 0.5 cm). Larger PCs are most common in young women [23]. In some cases, PC can cause hydrocephalus or Parinaud syndrome; here, surgical treatment is the gold standard.

However, patients with PCs can often present with nonspecific symptoms, including headache, sleep disturbances, dizziness, and nausea [1, 3, 7, 12, 17]. Patients with PCs are about twice as likely to suffer from headaches compared to patients in a control group without cysts [21]. The relationship between PC and these symptoms has not been conclusively established [2]. Previously, it was assumed that the headaches in these patients were due to increased intracranial pressure [4]. However, other studies suggest a hormonal imbalance, with melatonin thought to be the cause [15, 22]. Eide et al. [4] suggested that the severity of symptoms in patients with non-hydrocephalic PCs was related to compression of the internal cerebral veins causing central venous hypertension.

In case of unspecific symptoms, surgical treatment remains a matter of debate [1, 11]. According to a survey by Májovsky et al. [13], only 15% of the respondents occasionally operate on PCs with nonspecific symptoms. Nevertheless, in 42.9–100% there was an improvement of symptoms in patients after surgery—even with non-specific symptoms. Several surgical strategies are available to treat PCs: microsurgical, endoscopic, and stereotactic approaches. At our department, microsurgical fenestration is routinely performed to treat PCs. The aim of this study is to analyze the success rate of this surgical approach.

## Methods

This study was conducted in accordance with the 1964 Helsinki declaration and approved by the local ethics committee. Informed consent was obtained from all patients and/or their legal representatives.

### Patients

All patients undergoing microsurgical fenestration for PC from 2005–2021 were included in the analysis. Patients treated between 2005–2018 were retrospectively identified and contacted; this arm was combined with a prospective one, including all patients with PC treated at our institution between 2018–2021. A survey was conducted by a doctoral student with all patients. For the retrospectively included arm, the survey was conducted via telephone in April 2022. Exclusion criteria were: (a) Histological diagnosis differing from PC; and/or (b) conservative treatment only.

### Clinical parameters

Age at the time of surgery and sex were collected. Symptoms were classified as: (a) headaches; (b) dizziness; (c) double vision; (d) sleep irregularities; (e) others/unspecific. For headaches, their intensity was stratified according to the VAS from 0 (no pain) to 10 (worst headache). Symptom duration was also collected. Use of analgesics pre- and postoperatively was documented, including medications and dosage. These parameters were collected in a survey. Additionally, patients were asked how much their symptoms affected their quality of life preoperatively (slightly, moderately, severely), and whether surgery was able to alleviate them (no symptoms, improved symptoms postoperatively, no change in symptoms, symptoms worsened). The survey can be found as supplementary electronic material (SEM) 1.

### Radiographic parameters

Pre- and postoperative magnetic resonance imaging (MRI) was analyzed. The maximum anteroposterior and craniocaudal diameters of the PC were determined in sagittal and axial planes, as well as the presence of septation and/or contrast enhancement were analyzed. The presence of hydrocephalus was objectivized through the Evan’s index (ratio of the maximum width of the frontal horns of the lateral ventricles and the maximal internal diameter of the skull at the level of the third ventricle on axial imaging), CSF diapedesis was documented as well. Patency of the cerebral aqueduct was also documented.

### Surgical indication and strategy

Patients were considered candidates for PC fenestration if they had confirmed hydrocephalus on imaging. In cases where hydrocephalus was not present, PC fenestration was indicated when other causes for patient’s symptoms were ruled out by MRI and consultation (neurology, psychiatry, ophthalmology).

**Fig. 1:**
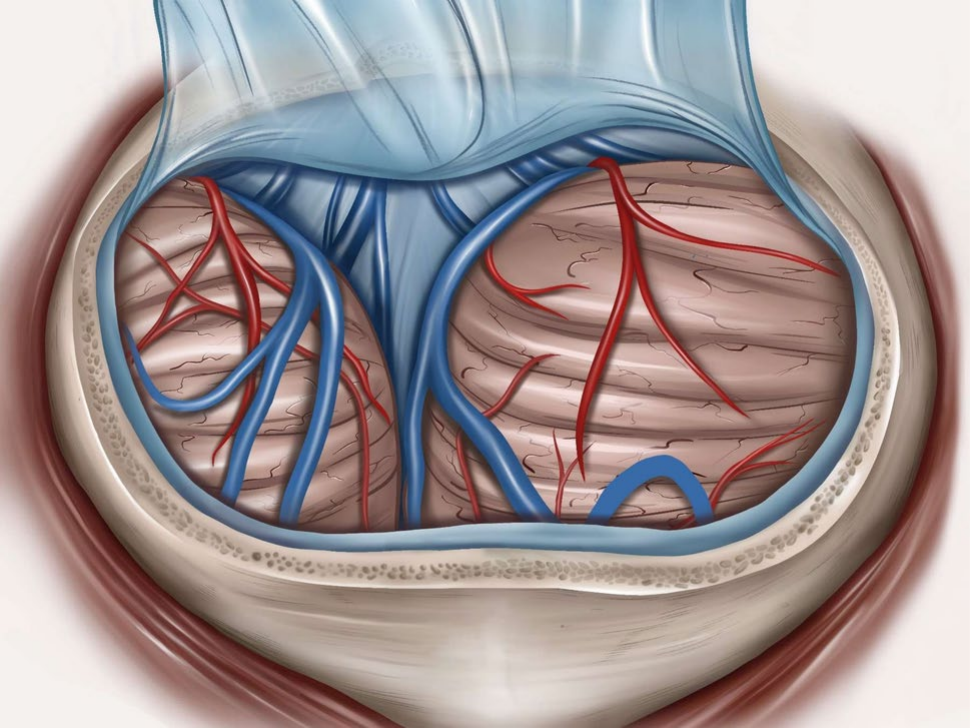
Median suboccipital craniotomy allowing for visualization of the superior surface of the vermis and cerebellar hemispheres after ligation of the occipital sinus (if present) and reflection of the dura cranially

**Fig. 2:**
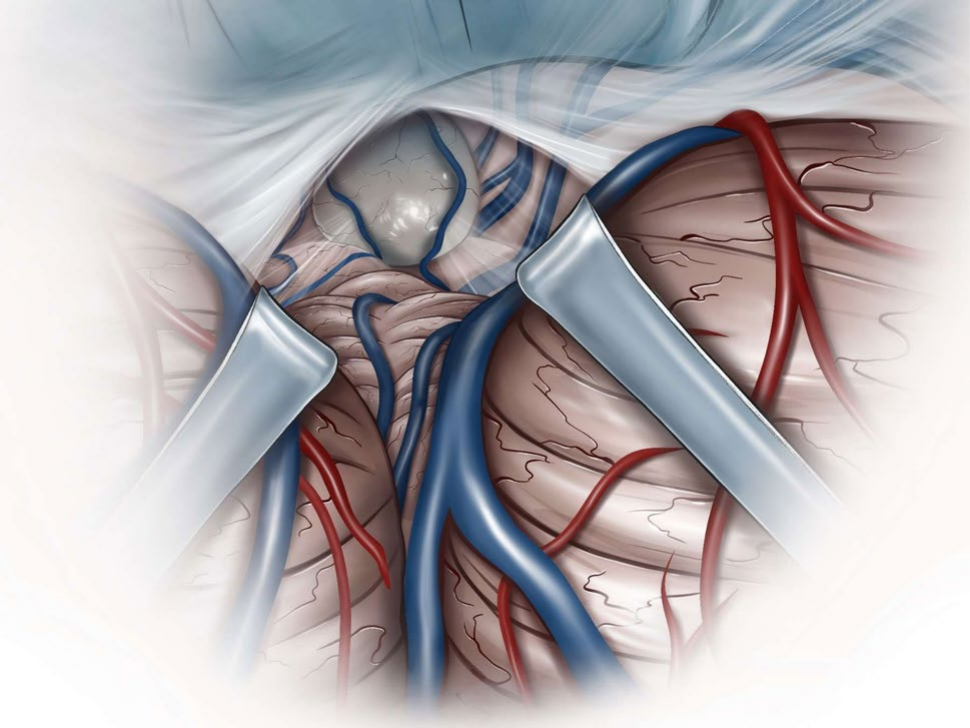
Through the use of retractors (here two exemplary ones, normally achievable through passive retraction or the use of a single one), caudal displacement of the superior cerebellar surface, leaving venous structures intact, if possible

**Fig. 3:**
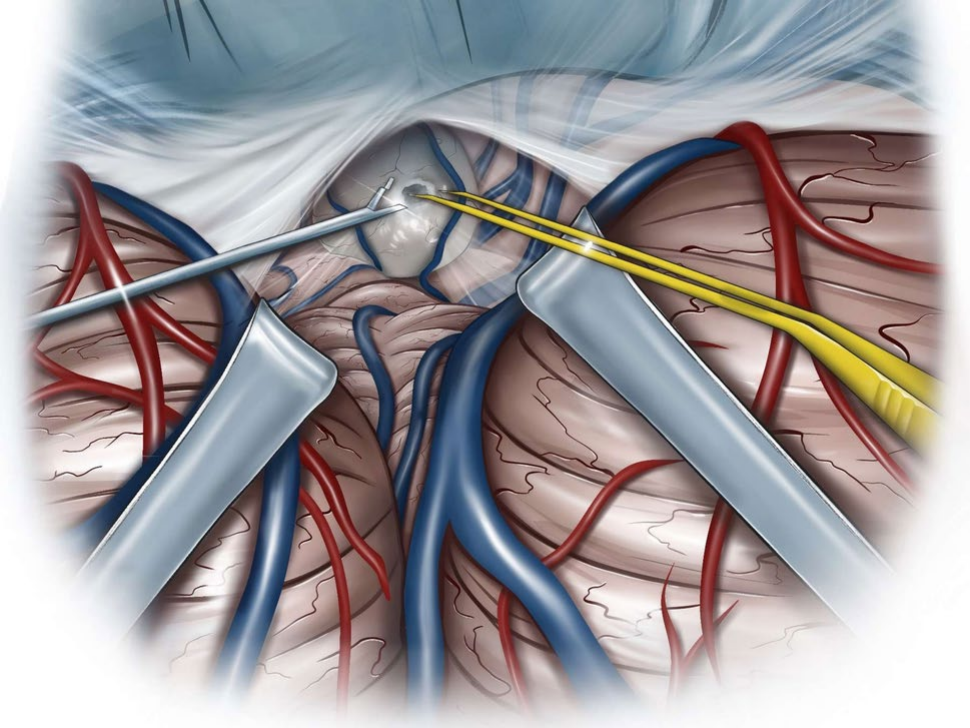
Through this corridor, visualization of the posterior wall of the pineal cyst, with the internal cerebral veins laterally and the Vein of Galen superiorly. Removal of a piece of the posterior wall of the cyst for histological assessment

**Fig. 4:**
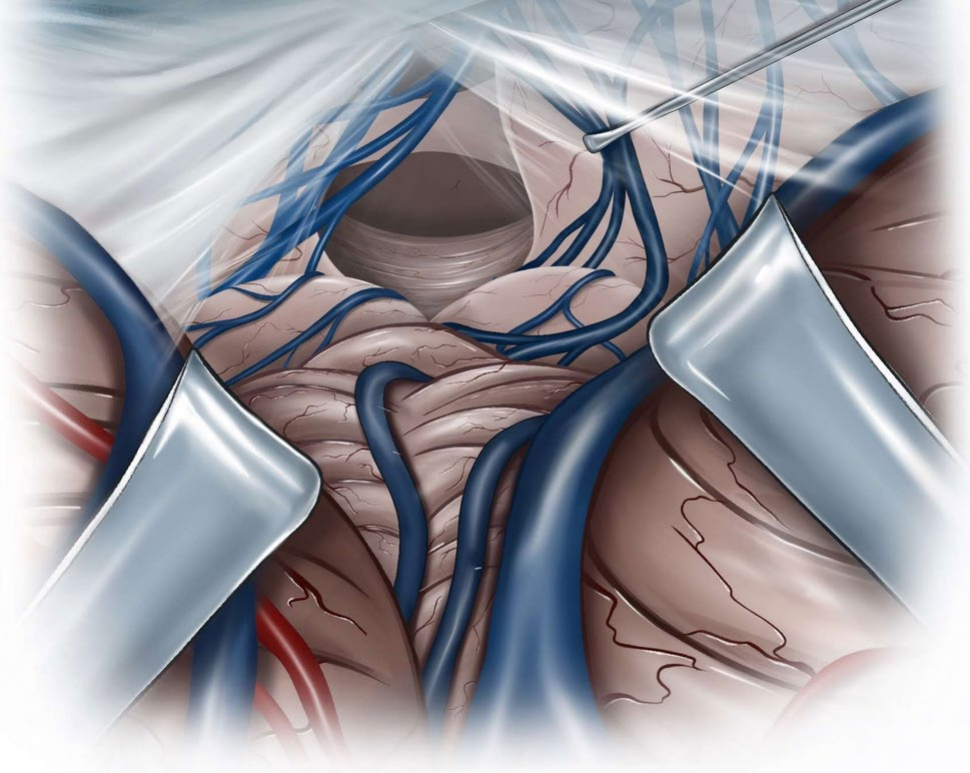
Sufficient incision at the anterior wall of the cyst allowing for visualization of the third ventricle and cerebrospinal fluid flow through the cyst

Microsurgical fenestration of the PC was performed via a midline suboccipital supracerebellar approach in all cases. Surgery was performed in sitting position after exclusion of a patent foramen ovale (PFO) through transesophageal echocardiography. In cases of PFO, patients were operated on in prone position. A midline incision was made, extending from approximately two centimetres above the inion to approximately two centimetres below the superior nuchal line. With a high-speed drill, one burr-hole was placed at the inion, and a median suboccipital craniotomy of approximately 3 cm in diameter was performed. After ligation of the occipital sinus (in case it was present), the dura was opened and reflected cranially, allowing for visualization of the superior surface of the vermis and cerebellar hemispheres (Fig. 1). Through the use of retractors, the superior cerebellar surface was displaced caudally; superior vermal veins were carefully dissected and, if possible, left intact. Through this corridor, the posterior wall of the PC was visualized, with the internal cerebral veins laterally and the Vein of Galen superiorly (Fig. 2). A sufficient incision was made at the posterior wall of the PC, and a piece of the posterior wall removed and sent to pathology for histological assessment (Fig. 3). Then, a sufficient incision was made at the anterior wall of the PC until visualization of the third ventricle. CSF flow through the PC was thus obtained (Fig. 4). Once this fenestration was completed and hemostasis performed, the surgical site was closed in layers and the patient transferred to the intensive care unit for postoperative monitoring.

### Primary and secondary endpoints

The primary endpoint of this study was symptom improvement at last follow-up, as assessed by our survey. Radiographic and clinical factors were evaluated for their correlation with symptom improvement. Secondary endpoints were surgical complications, such as surgical site infections (SSI), CSF fistula, and postoperative hematoma.

### Statistical analysis

Means and standard deviations (SD) were used to report continuous variables. Frequencies and percentages were used to report categorical and ordinal variables. To assess symptom improvement at last follow-up, a paired-sample t-test was performed. Factors contributing to symptom improvement were assessed by means of Spearmann’s Rho correlation index. Statistical significance was assumed at *p* < 0.05.

## Results

A total of 58 patients were identified; of these, 47 patients were included in the analysis: 34 were retrospectively analyzed, while 13 were recruited prospectively, 11 patients declined to participate in the study. Table 3 shows the number of included patients per treatment year.

More than half of the patients were female (*n* = 29, 62%) and mean age was 23 years (SD 12.9). In our cohort, approximately a third (*n* = 17/47, 36%) were pediatric patients. In this subgroup, sex distribution was almost equal (*n* = 9/17, 53% female, *n* = 8/17, 47% male). On the contrary, in the adult subgroup, there was a female predominance (*n* = 20/30, 67%).

### Clinical parameters and surgical indication

Almost all patients reported preoperative headaches (*n* = 44/47, 94%). Nearly half experienced dizziness (*n* = 19/47, 49%) or double vision (*n* = 21/47, 45%), and *n* = 10/47, 21% of the patients had sleep disturbances. Among other symptoms, the patients reported nausea (*n* = 15/47, 32%), light sensitivity/visual disturbances (excluding double vision) (*n* = 9/47, 19%), difficulty concentrating/fatigue (*n* = 4/47, 9%), and sensory disturbances (*n* = 3/47, 6%). More than half of the patients (*n* = 26/47, 55%) reported experiencing severe daily impairment due to their symptoms. Only *n* = 8/47, 18% of the patients stated that their everyday activity was only slightly (*n* = 4/47, 9%) or not at all (*n* = 4/47, 9%) limited by the symptoms of the PCs. Nearly all of the surveyed patients (*n* = 46/47, 98%) viewed the surgical intervention as the last resort for their problems and therefore opted for the surgery.

### Radiological parameters

The majority (*n* = 32/47, 67%) of PC were small in diameter, spanning < 1.5 cm. The maximum diameter of the pineal body (pineal tissue + cyst(s)) was: mean 1.29 cm, minimum 0.5 cm, maximum 2.6 cm (Table 1). Solely one (*n* = 1/47, 2%) patient showed an increased Evan’s index of 0.3 and aqueduct occlusion on imaging, thus being the only patient in our cohort with mild hydrocephalus. Five patients showed CSF diapedesis but a normal Evan´s index. Over half of our cohort had PC septations (*n* = 26/47, 55%) and contrast-enhancement (*n* = 24/47, 51%). 27 patients showed a narrowing of the aqueduct, but with flow void present. There was no venous edema present in any cases.

**Table 1:** Dimensions of the pineal body (=pineal tissue + cyst(s)) in preoperative magnetic resonance imaging

Dimension	Minimum	Maximum	Mean	Standard deviation
Coronal (in cm)	0.4	2.0	1.03	0.33
Sagittal (in cm)	0.5	2.6	1.29	0.40
Axial (in cm)	0.5	1.8	1.04	0.31
Volume (in cm^3^)			0.83	0.65

Postoperative MRI was only performed in the case of symptoms or complications (*n* = 9). Four patients showed a decrease in the size of the PC. Regarding the other five patients, no statement can be made about the size of the cyst in the postoperative MRI, as only the written report was available. No further new pathological findings were observed in any of the cases. The timing of postoperative MRI was heterogeneous, ranging from days to several years after surgery.

### Endpoints

The patient survey was conducted in April 2022. The follow-up period ranged from 5 to 176 months, with an average follow-up time of 84.13 months (about 7 years) post-surgery. Mean preoperative VAS was 7 (SD 2.34), while postoperatively it was 1 (SD 1.91). The majority of patients (*n* = 44/47, 94%) showed a decrease in their VAS postoperatively. In a paired-sample t-test for the VAS, the postoperative improvement was statistically significant (*p* < 0.001). This was confirmed by patients in the survey, as *n* = 30/47, 62% reported being asymptomatic after surgery, while *n* = 17/47, 36% reported being significantly better. Most patients were unable to remember the medication they precieved postoperatively due to the sometimes long period of time since surgery. The medication documented at discharge could also not be assessed because of bias due to wound pain, which is often not insignificant in the case of suboccipital approch due to muscle damage. For analysing pain medication we therefore focused on the the last follow- up (April 2022). Over half of the patients (*n* = 27/47, 57%) reported use of non-steroidal anti-inflammatory drugs (NSAIDs), and *n* = 8/47, 17% reported use of opiates prior to surgery. These percentages were reduced to *n* = 15/47, 31% and *n* = 1/47, 2% at the last follow-up, respectively. In the subgroup of patients who were still symptomatic, the use of NSAIDs was reduced from 13 to 11 patients, which was not significant due to paired t-test (*p* = 0.3299). Opiate use was reduced from four patients to one patient, which was also not significant (*p* = 0.0856).

Among the preoperative variables analyzed, none correlated with clinical improvement, as summarized in Table 2. Furthermore, no mortality or haemodynamically relevant intraoperative bleeding or injury to the internal cerebral veins were observed. CSF leaks were observed in *n* = 5/47, 10% of patients; these were all treated conservatively with a lumbar drain. SSI were observed in only *n* = 1/47 (2%) of cases.

**Table 2:** Preoperative variables analysed for the prediction of symptom improvement after microsurgical pineal cyst fenestration

Preoperative Symptoms, Medication and radiological parameters	Percentage or mean and standard deviation	*P* value (Spearman´s correlation)
Headache	94 %	.649
Dizziness	49 %	.083
Visual disturbances	45 %	.691
Sleep disturbances	21 %	.607
VAS preoperatively	Mean 7 (SD 2.34)	.061
Symptom duration in months	Mean 13 (SD 11.48)	.829
Impaired quality of life Limitation of daily activities	92 %	.339
Preoperative use of NSAIDs	57 %	.649
Preoperative use of opiates	17 %	.431
Cyst volume in cm³	Mean.84 (SD.66)	.693
Septations present	55 %	.440
Contrast enhancement	51 %	.586
Hydrocephalus	2 %	.797

NSAID- non-steroidal anti-inflammatory drug; VAS – visual analogue scale

## Discussion

This case series is comparatively one of the largest cohorts of PC patients with specific symptoms undergoing microsurgical cyst fenestration with significant clinical improvement. There are two main issues that we would like to discuss on the basis of our data. The first is the unclear indication for surgery to date, and the second is the question of surgical technique – resection vs. fenestration of the PC.

### Surgical technique

We consider fenestration to be a safer treatment option than cyst resection, as most PCs have a close relationship to the internal cerebral veins and the vein of Galen. Complete resection of the PC might be associated with an increased intraoperative bleeding risk and resection of or damage to the often flattened and pushed sidewards PG, which can result in morbidity and mortality. Nevertheless, this surgical strategy appears to be controversial in the literature.

According to a meta-analysis performed by Masina et al., most patients with PC are treated by cyst resection (*n* = 252), and only a few (*n* = 9) by fenestration. In this pooled analysis, fenestration achieved worse outcomes than cyst resection [16]. However, due to the small number of patients undergoing fenestration, these results must be interpreted with caution, as they are subject to type II error. Similarly, a literature review by Majovski et al. that evaluated 10 studies between 1991 and 2017 on surgical treatment of PCs included only one study on patients who received cyst fenestration[14]:

In a population of 27 cases, Eide and Ringstad reported that complete microsurgical resection of the PC (*n* = 15 patients) led to symptom relief in all patients, 93% of the patients reported significant symptom relief, while microsurgical cyst fenestration only led to slight symptom relief in 67% (*n* = 6) of all patients and no patients reported significant symptom relief [5]. Contrarily, we achieved an improvement rate of 96% by cyst fenestration, which is comparable to other studies in which resection was preferred [4, 5, 11, 14].

We attribute the high rate of success in our patient cohort to the fenestration of both the anterior and posterior cyst walls with exposure of the third ventricle. In the case of incomplete fenestration, drainage of the cyst fluid cannot be fully guaranteed, resulting in residual or persistent symptoms. Since we have no control cohort with only posterior PC windowing – which would be ethically questionable – we obviously cannot prove this statistically. Further pathophysiological studies on PC might provide a more detailed explanation as to why patients benefited from fenestration.

### Indication for surgery

The benefits of surgical treatment per se have been increasingly confirmed in recent years. Until the first large cohort of patients with non-hydrocephalic PC published by Kalani et al. in 2015, there were only case reports suggesting a weak association between resection of the cyst and improvement in symptoms [11]. Since then, several clinical series have reported similar results [4, 6, 8, 14]. Májovský et al. reported on a series of 21 patients who underwent PC resection [14]. Twenty patients (95.2%) in this series reported an improvement in their symptoms, with 10 of these patients (47.6%) being symptom-free after surgery. These results are comparable to ours, thus strengthening the body of evidence in favor of surgery for patients with PC symptomatic pineal cyst syndrome.

In our patients, 96% experienced symptom relief after surgery. For improvement of headache on VAS, this improvement was statistically significant. The long-term success in most of our patients, as well as the improvement in our paediatric cohort, makes a placebo effect unlikely (follow-up period = 5–176 months) [24].

While we were able to achieve significant clinical improvement in our patients, none of the variables analyzed correlated with clinical improvement. For instance, cyst volume did not appear to be predictive of symptom improvement after surgery. The correlation between cyst size and symptomatology remains a subject of debate. Although symptomatic PCs are typically larger than 2 cm, cases with symptomatic cysts as small as 0.7 cm have been documented in the literature. Kalani et al. [11] demonstrated postoperative symptom improvement in 17 of 18 patients with PCs that did not involve hydrocephalus or tectal compression, with a mean cyst diameter of 1.5 cm. These findings suggest that patients with small PCs with nonspecific symptoms are not necessarily poor candidates for surgical intervention and aligns with our observations.

Similarly, we were not able to prove an association between radiological appearance and symptom improvement. In our study, 55% of cysts had one or more septa. In a study by Nolte et al., 41% of PC also had septations. In fact, high-resolution MRI studies have shown that internal septations could be detected in the vast majority of PCs [18]. The fact that patients with an anatomically complex PC with multiple septa had as much of a clinical improvement after surgery as those without suggests that complex radiological appearance does not preclude good surgical candidacy.

As there are no clear indications in the literature regarding the need for surgical treatment of non-hydrocephalic PC [6, 14, 25] and we failed to identify clinical and/or radiological parameters that could indicate better surgical candidacy, we advocate for careful selection of patients for surgery, as even patients with small cysts can experience a significant clinical improvement.

### Limitations and future directions

In our cohort, we did not evaluate the melatonin levels of our patients. There is increasing evidence that headache disorders are related to melatonin secretion and PG function, and both migraine and cluster headaches have been shown to have reduced melatonin levels [19]. A prospective study on melatonin levels and PC is currently being conducted at our centre.

Another potential bias of this study is a recall bias, as patients were interviewed at differing time points postoperatively (Table 3). Furthermore, we did not include a control arm with patients being treated conservatively, and/or by means of surgical excision of the cyst. A randomized controlled trial of non-hydrocephalic patients with symptomatic PC could shed some light on whether or not surgery truly is a superior treatment alternative to medication alone. In addition to evaluating the optimal surgical technique, selection criteria for suitable patients must be developed and the pathophysiology of symptomatic PC syndrome must be worked out. The most recent literature in this regard deals with MRI parameters that indicate venous outflow obstruction [20] and also with disturbed melatonin secretion. This necessity is further reinforced by a patient survey in which 72.1% of conservatively treated patients reported no significant improvement in symptoms [10].

**Table 3:** This data is mandatory

Treatment year	Number of cases per year
2007	4
2008	2
2009	2
2010	5
2011	5
2012	7
2013	2
2014	4
2015	5
2016	5
2017	4
2018	2
2019	3
2020	4
2021	4

* indicates that from here on, prospective study inclusion took place

## Conclusion

Microsurgical cyst fenestration can yield symptom improvement in non-hydrocephalic patients with symptomatic PC syndrome. Further work is needed to validate both this surgical technique and indications therefor.

## Supplementary Information

Below is the link to the electronic supplementary material.

Supplementary file1 (PDF 58 KB)

## Data Availability

The results of the survey can be requested from the corresponding author.

## Abbreviations

CSF: Cerebrospinal fluid
MRI: Magnetic resonance imaging
NSAID: Non-steroidal anti-inflammatory drugs
PC: Pineal cyst
PG: Pineal gland
PFO: Patent foramen ovale
PG: Pineal gland
SD: Standard deviation
SEM: Supplementary electronic material
SSI: Surgical site infection
VAS: Visual analogue scale

## Declarations

### Previous presentation of data

The results of this study were presented in advance at the Annual Meeting of the German Society for Neurosurgery in Stuttgart, June 2023. Parts of the results will be published in German language by the University of Göttingen as part of a doctoral thesis.

## References

[1] Al-Holou WN, Terman SW, Kilburg C, Garton HJ, Muraszko KM, Chandler WF, Ibrahim M, Maher CO (2011) Prevalence and natural history of pineal cysts in adults. J Neurosurg 115:1106–1114. 10.3171/2011.6.Jns11506 21780858 10.3171/2011.6.JNS11506

[2] Barboriak DP, Lee L, Provenzale JM (2001) Serial MR imaging of pineal cysts: implications for natural history and follow-up. AJR Am J Roentgenol 176:737–743. 10.2214/ajr.176.3.1760737 11222216 10.2214/ajr.176.3.1760737

[3] Choy W, Kim W, Spasic M, Voth B, Yew A, Yang I (2011) Pineal cyst: a review of clinical and radiological features. Neurosurg Clin N Am 22(341–351):vii. 10.1016/j.nec.2011.06.001 21801982 10.1016/j.nec.2011.06.001

[4] Eide PK, Ringstad G (2016) Increased pulsatile intracranial pressure in patients with symptomatic pineal cysts and magnetic resonance imaging biomarkers indicative of central venous hypertension. J Neurol Sci 367:247–255. 10.1016/j.jns.2016.06.028 27423599 10.1016/j.jns.2016.06.028

[5] Eide PK, Ringstad G (2017) Results of surgery in symptomatic non-hydrocephalic pineal cysts: role of magnetic resonance imaging biomarkers indicative of central venous hypertension. Acta Neurochir (Wien) 159:349–361. 10.1007/s00701-016-3029-4 27878615 10.1007/s00701-016-3029-4

[6] El Damaty A, Fleck S, Matthes M, Baldauf J, Schroeder HWS (2019) Pineal Cyst without Hydrocephalus: Clinical Presentation and Postoperative Clinical Course After Infratentorial Supracerebellar Resection. World Neurosurg 129:e530–e537. 10.1016/j.wneu.2019.05.200 31154104 10.1016/j.wneu.2019.05.200

[7] Evans RW, Peres MF (2010) Headaches and pineal cysts. Headache 50:666–668. 10.1111/j.1526-4610.2010.01652.x 20456152 10.1111/j.1526-4610.2010.01652.x

[8] Fedorko S, Zweckberger K, Unterberg AW (2018) Quality of life following surgical treatment of lesions within the pineal region. J Neurosurg 130:28–37. 10.3171/2017.7.Jns17260 29498568 10.3171/2017.7.JNS17260

[9] Golzarian J, Balériaux D, Bank WO, Matos C, Flament-Durand J (1993) Pineal cyst: normal or pathological? Neuroradiology 35:251–253. 10.1007/bf00602604 8492885 10.1007/BF00602604

[10] Harding J, Masina R, Hill A, Ansanipour A, Steele A, Kolias A, Santarius T (2024) International web-based survey of patients with non-hydrocephalic symptomatic pineal cysts. Acta Neurochir (Wien) 166:509. 10.1007/s00701-024-06403-5 39731656 10.1007/s00701-024-06403-5 PMC11682015

[11] Kalani MY, Wilson DA, Koechlin NO, Abuhusain HJ, Dlouhy BJ, Gunawardena MP, Nozue-Okada K, Teo C (2015) Pineal cyst resection in the absence of ventriculomegaly or Parinaud’s syndrome: clinical outcomes and implications for patient selection. J Neurosurg 123:352–356. 10.3171/2014.9.Jns141081 25932610 10.3171/2014.9.JNS141081

[12] Lacroix-Boudhrioua V, Linglart A, Ancel PY, Falip C, Bougnères PF, Adamsbaum C (2011) Pineal cysts in children. Insights. Imaging 2:671–678. 10.1007/s13244-011-0117-0 10.1007/s13244-011-0117-0 PMC3259367 22347985

[13] Májovský M, Netuka D, Beneš V (2016) Clinical management of pineal cysts: a worldwide online survey. Acta Neurochir (Wien) 158:663–669. 10.1007/s00701-016-2726-3 26897024 10.1007/s00701-016-2726-3

[14] Májovský M, Netuka D, Beneš V (2018) Is surgery for pineal cysts safe and effective? Short review. Neurosurg Rev 41:119–124. 10.1007/s10143-017-0876-2 28702847 10.1007/s10143-017-0876-2

[15] Mandera M (2003) Usefulness of melatonin in the diagnostics and therapy of pineal gland and brain neoplasms. Wiad Lek 56:569–573 15058167

[16] Masina R, Ansaripour A, Beneš V, Berhouma M, Choque-Velasquez J, Eide PK, Fedorko S, Fleck S, Hernesniemi J, Koziarski A, Májovský M, Podgorski A, Schroeder H, Teo C, Unterberg AW, Yeung JT, Kolias A, Santarius T (2022) Surgical treatment of symptomatic pineal cysts without hydrocephalus-meta-analysis of the published literature. Acta Neurochir (Wien) 164:61–77. 10.1007/s00701-021-05054-0 34854993 10.1007/s00701-021-05054-0 PMC8761144

[17] Mena H, Armonda RA, Ribas JL, Ondra SL, Rushing EJ (1997) Nonneoplastic pineal cysts: a clinicopathologic study of twenty-one cases. Ann Diagn Pathol 1:11–18. 10.1016/s1092-9134(97)80004-4 9869821 10.1016/s1092-9134(97)80004-4

[18] Nolte I, Brockmann MA, Gerigk L, Groden C, Scharf J (2010) TrueFISP imaging of the pineal gland: more cysts and more abnormalities. Clin Neurol Neurosurg 112:204–208. 10.1016/j.clineuro.2009.11.010 20034731 10.1016/j.clineuro.2009.11.010

[19] Peres MF (2005) Melatonin, the pineal gland and their implications for headache disorders. Cephalalgia 25:403–411. 10.1111/j.1468-2982.2005.00889.x 15910564 10.1111/j.1468-2982.2005.00889.x

[20] Santarius T, Pickard JD (2023) Does deep cerebral venous engorgement contribute to non-hydrocephalic pineal cysts becoming symptomatic? Some missing links. Brain Commun 5:fcad096. 10.1093/braincomms/fcad096 37065089 10.1093/braincomms/fcad096 PMC10090880

[21] Seifert CL, Woeller A, Valet M, Zimmer C, Berthele A, Tölle T, Sprenger T (2008) Headaches and pineal cyst: a case-control study. Headache 48:448–452. 10.1111/j.1526-4610.2007.00965.x 18005138 10.1111/j.1526-4610.2007.00965.x

[22] Starke RM, Cappuzzo JM, Erickson NJ, Sherman JH (2017) Pineal cysts and other pineal region malignancies: determining factors predictive of hydrocephalus and malignancy. J Neurosurg 127:249–254. 10.3171/2016.8.Jns16220 27767399 10.3171/2016.8.JNS16220

[23] Warsza B, Due-Tønnessen P, Due-Tønnessen P, Pripp A, Ringstad G, Eide PK (2023) Prevalence of pineal cysts in healthy individuals: Emphasis on size, morphology and pineal recess crowding. J Neurol Sci 453:120801. 10.1016/j.jns.2023.120801 37741123 10.1016/j.jns.2023.120801

[24] Weimer K, Gulewitsch MD, Schlarb AA, Schwille-Kiuntke J, Klosterhalfen S, Enck P (2013) Placebo effects in children: a review. Pediatr Res 74:96–102. 10.1038/pr.2013.66 23598811 10.1038/pr.2013.66

[25] Wisoff JH, Epstein F (1992) Surgical management of symptomatic pineal cysts. J Neurosurg 77:896–900. 10.3171/jns.1992.77.6.0896 1432132 10.3171/jns.1992.77.6.0896

