# Supplementary material for: Microsurgical pineal cyst fenestration: A safe and effective treatment strategy in patients with symptomatic pineal cyst syndrome
Source: Acta Neurochir (Wien). 2025 Jun 30;167(1):179. doi: 10.1007/s00701-025-06569-6 (PMC12208953; doi:10.1007/s00701-025-06569-6)
Supplement: Supplementary file 1 — Supplementary file1 (PDF 58 KB) [file 701_2025_6569_MOESM1_ESM.pdf]

PATIENT SURVEY

1. Which symptoms did you have before surgery? (check all that apply)

   - ☐ Dizziness
   - ☐ Headaches
   - ☐ Double vision
   - ☐ Sleep disturbances
   - ☐ Other: ________________________

2. How long did you have these symptoms before surgery?

   ______ years ______ months

3. How much did these symptoms affect your daily life before surgery?

   - ☐ Severely
   - ☐ Moderately
   - ☐ Mildly
   - ☐ Not at all

4. How strong were your symptoms? (0= no symptoms, 10= most severe symptoms)

   Before surgery:

   0 1 2 3 4 5 6 7 8 9 10

   Immediately after surgery:

   0 1 2 3 4 5 6 7 8 9 10

   Currently:

   0 1 2 3 4 5 6 7 8 9 10

5. Did your symptoms improve after surgery?

☐ no symptoms
☐ improved
☐ no change
☐ worsened

6. Was surgery your last resort?
   ☐ yes
   ☐ no

7. Were there any complications after surgery? If yes, which?

   ☐ no
   ☐ yes, the following: ___________________________

8. How long did it take you to resume your normal daily activities after surgery?

   ______weeks ______months

9. Would you recommend surgery to patients with similar symptoms?

   ☐ yes
   ☐ no

10. Would you undergo surgery again?
    ☐ yes
    ☐ no

11. Which pain killers did you take and how often? (Check all that apply)

		Daily	A few times a week	A few times a month	Seldomly
**Before** surgery:	Ibuprofen				
	Paracetamol				
	Metamizole				
	Acetyl-salicylic acid (Aspirin®)				
	Opiate derivates				
	Other:				

		Daily	A few times a week	A few times a month	Seldomly

**Immediately after** surgery:	Ibuprofen				
	Paracetamol				
	Metamizole				
	Acetyl-salicylic acid (Aspirin®)				
	Opiate derivates				
	Other:				

		Daily	A few times a week	A few times a month	Seldomly
**Currently:**	Ibuprofen				
	Paracetamol				
	Metamizole				
	Acetyl-salicylic acid (Aspirin®)				
	Opiate derivates				
	Other:				
